# MD/DO-PhD trainees: Flexing, adapting, and progressing during COVID-19

**DOI:** 10.1017/cts.2020.559

**Published:** 2020-11-23

**Authors:** Gina Calco, Hanna Erickson, Omar Toubat, Samantha Spellicy

**Affiliations:** 1 MD/PhD Program, Division of Pulmonary and Critical Care Medicine, Oregon Health & Science University, Portland, OR, USA; 2 American Physician Scientists Association, Chicago, IL, USA; 3 MD/PhD Program, University of Illinois College of Medicine at Urbana, Urbana, IL, USA; 4 MD/PhD Program, Department of Surgery, Keck School of Medicine of USC-California Institute of Technology, Los Angeles, CA, USA; 5 MD/PhD Program, Office of Academic Affairs, Medical College of Georgia, University System of Georgia, Augusta, GA, USA

**Keywords:** Physician–Scientist, COVID-19, pandemic, clinical training, graduate training

## Introduction

For MD/DO-PhD trainees, who pursue an extended training pathway with formal learning in both clinical and research settings, the COVID-19 pandemic has brought a unique set of educational challenges. In particular, the pandemic has added strain on the already challenging transitions from medical school to graduate training and back. This is on top of the impact felt more broadly by medical and graduate trainees. Such a strain may increase attrition in an already leaky physician–scientist workforce pathway [1]. Here, we provide a trainee perspective outlining the effects the pandemic has had on the various stages of the predoctoral dual-degree training paradigm and highlight opportunities for intervention to aid MD/DO-PhD trainees.

## General Trainee Concerns

At the start of the pandemic, there were radical shifts in medical training and research. For preclinical medical students, all lectures became virtual and the few clinical experiences during this time were either modified or canceled altogether. Similarly, students in their clerkship years were taken out of the clinic and moved to virtual didactics. Although medical students have largely returned to in-person clerkships, their learning experience continues to be impacted by shortened rotation schedules and restricted access to patients. Additionally, medical licensing exams were delayed for many students due to test center closings.

On the research side, many academic research laboratories were required to shut down and effectively cease research activity [2]. At many institutions, animal colonies were forcibly downsized, and experiments were delayed as physical access to lab space was restricted. Students further along in their research training also experienced publication delays and subsequent uncertainty around scheduling dissertation defenses and completing other graduate degree requirements [3]. Given the persistent limitations on occupancy and restricted access to core facilities and laboratories, productivity for many trainees in research remains significantly reduced.

## Specific Concerns for MD/DO-PhD Trainees

**Fig. 1.**
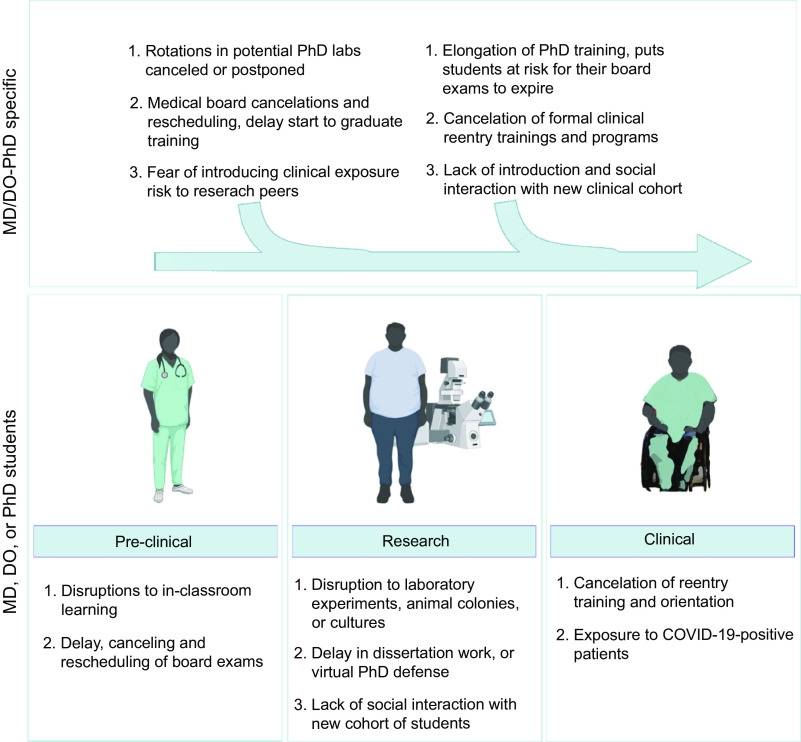
Detrimental effect of the COVID-19 pandemic on MD/DO-PhD training. Graphic depicting specific hurdles and difficulties the COVID-19 pandemic has presented to MD/DO-PhD students (top row) specifically in addition to those encountered by their MD-, DO-, or PhD-only peers (bottom row).

The impact of COVID-19 on research productivity and PhD completion presents a unique concern for MD/DO-PhD students due to the strict educational timeline assigned by most dual-degree programs (Fig. 1). In general, these programs split the medical school curriculum after the second or third year of medical training (and completion of the United States Medical Licensing Exam (USMLE) Step 1) and place a limitation of 4–5 years for PhD research. The rationale for this timeline is to ensure progression through the program and to accommodate medical licensure time limits assigned by the state medical boards, of which some require that the USMLE Steps 1–3 exams be completed in a 7-year period. As a consequence, many MD/DO-PhD students have faced a dilemma: finish their PhD or finish their medical training within the time allotted.

Another sequela of the pandemic is decreased preparedness of MD/DO-PhD students as they transition back into the clinic. While most institutions typically utilize reentry modules or courses to help students refamiliarize themselves with medicine, many of these programs were halted or canceled in the wake of the pandemic. As a result, many students transitioned into clerkships without a chance to practice and re-hone their clinical skills. These courses are particularly important for MD/DO-PhD trainees, who have been out of the clinical environment for 3 or more years. Furthermore, MD/DO-PhD students reenter their medical training with a new medical school class. With the cancelation of many in-person social events, transitioning MD/DO-PhD trainees have few remaining opportunities to develop meaningful connections with new classmates. This can negatively impact mental health due to increased isolation and decreased social interaction [4–6].

There is a similar effect on the transition from medical school to graduate training. Scheduling delays for USMLE Step exams can delay the start of graduate training. Furthermore, workspace restrictions and decreased research activity have curtailed opportunities for new graduate students to conduct research rotations. Additionally, the lack of in-person social events has impacted the ability to form supporting connections with new classmates and labmates.

## Recommendations

According to the physician–scientist workforce report (2014), individuals with MD/DO-PhD training comprise 45% of all NIH-funded physician–scientists. While these degrees can be pursued separately, a common training pathway is to pursue these degrees through a combined MD/DO-PhD program. To maintain the physician–scientist workforce pathway, it is essential that training programs and professional organizations adapt to best support trainees through these challenging times.

The COVID-19 pandemic has forced MD/DO-PhD trainees to make difficult decisions about their clinical and research training, including delaying graduation at the risk of financial and medical licensing impacts or forgoing research training altogether. At this time, MD/DO-PhD programs need to be flexible with their timeline to allow MD/DO-PhD students adequate time to complete their PhDs without an impact on their stipend and degree standing. Furthermore, state medical boards should consider extending their time limit to complete USMLE Steps 1–3 to at least 10 years to accommodate for MD/DO-PhD trainees [7].

Additionally, there is a need for education and support during times of transition for MD/DO-PhD trainees. Virtual platforms have now replaced traditional social gatherings, providing space for MD/DO-PhD students to adjust and get to know their peers during transitions. Furthermore, organizations such as the American Physician Scientists Association (APSA) have continued virtual advocacy efforts for physician–scientist trainees throughout the pandemic. APSA has hosted virtual interactive sessions and panel discussions for trainees at all levels, including MD/DO-PhD program information sessions and residency information sessions. Importantly, APSA has also hosted a series of virtual interactive sessions for dual-degree applicants that identify as underrepresented minorities. However, there is a still need for virtual resources to help prepare MD/DO-PhD students for transitioning to research rotations and clinical training.

Overall, the COVID-19 pandemic has had an impact on every stage of the dual-degree physician–scientist training pathway. We recognize that the impact of the pandemic will vary based on the stage of training, training location, and personal factors including family status and finances. Thus, there is a need for comprehensive data to assess the impact of the pandemic across the spectrum of MD/DO-PhD training experiences. Moving forward, it is critical to continue optimizing these efforts in order to support and sustain the next generation of physician–scientists.
